# Food and Nutrition Public Policies in Brazil: From Malnutrition to Obesity

**DOI:** 10.3390/nu14122472

**Published:** 2022-06-15

**Authors:** Ligia Moriguchi Watanabe, Heitor Bernardes Pereira Delfino, Marcela Augusta de Souza Pinhel, Natália Yumi Noronha, Luisa Maria Diani, Lucca Cintra do Prado Assumpção, Carolina Ferreira Nicoletti, Carla Barbosa Nonino

**Affiliations:** 1 Department of Health Sciences, Ribeirao Preto Medical School, University of Sao Paulo, Ribeirão Preto 14049-900, Brazil; assumpcao.jandyra@usp.br (L.C.d.P.A.); carla@fmrp.usp.br (C.B.N.); 2 Department of Internal Medicine, Ribeirao Preto Medical School, University of Sao Paulo, Ribeirão Preto 14049-900, Brazil; heitorbernardes@usp.br (H.B.P.D.); marcelapinhel@yahoo.com.br (M.A.d.S.P.); natty.yumi@gmail.com (N.Y.N.); luisa.diani@usp.br (L.M.D.); 3 Department of Molecular Biology, Sao Jose do Rio Preto Medical School, Sao Jose do Rio Preto 15090-000, Brazil; 4 Applied Physiology & Nutrition Research Group, Rheumatology Division, Medical School FMUSP, University of Sao Paulo, Sao Paulo 01246-903, Brazil; carol_nicolettif@yahoo.com.br

**Keywords:** nutritional transition, food, nutrition, public policies, Brazil

## Abstract

“Nutrition transition” describes the shifts in dietary consumption and energy expenditure influenced by economic, demographic, and epidemiological changes at a population level. This phenomenon has been associated with rising obesity rates worldwide, especially in developed countries. In Brazil, the historical analysis of temporal trends between malnutrition and obesity characterized the nutrition transition in the country and interweaved it with the formulation and implementation of public food and nutrition policies. Such analysis is crucial for understanding certain principles in each context. Thus, this review contextualized the consolidation of obesity as a critical health and public policy issue in Brazil. Our review suggested that the country may still be at the initial stage of care for obesity, and more efforts are needed to contain the advance of the disease in Brazil.

## 1. Introduction

The “transition” concept has been widely applied in many research areas to display significant trends and further understand the population health parameters [1]. “Nutrition transition” has emerged as an important concept in health research used to describe shifts in dietary consumption and energy expenditure that coincide with economic, demographic, and epidemiological changes at a population level [2]. Although nutrition transition is a worldwide social problem, this term has been predominantly used to denote the transitions observed in developing countries from traditional diets to diets containing mainly processed/ultra-processed foods [1,2]. This shift in dietary patterns has been found to largely coincide with rising rates of obesity, contributing to the increase in related diseases such as diabetes, non-alcoholic fatty liver disease, metabolic syndrome, and other metabolic diseases [3,4], bringing a new scenario in terms of morbidity and mortality in most regions of the planet [5].

In Brazil, from the analysis of the historical context in the last 50 years, it was possible to observe an antagonism of temporal trends between malnutrition and obesity that were influenced by demographic transition and culminated in the occurrence of the nutrition transition and epidemiological transition in the country [6,7]. Following this line, the analysis of the landmarks of the trajectory of formulating and implementing public food and nutrition policies in Brazil could be situated within an approach that comprises three aspects: availability of food, its consumption, and, finally, biological use of energy and nutrients that reflects the state of nutrition in the Brazilian population [7].

**Figure A1:**
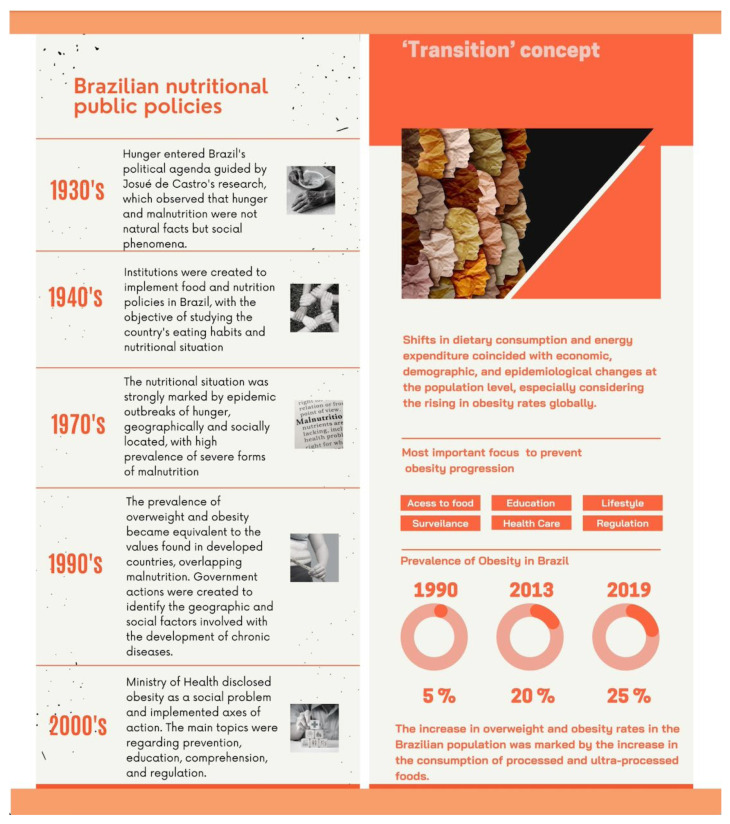
The historical progress of the nutrition transition in Brazil and the trajectory of Brazilian nutritional public policies.

This review disclosed the historical progress of the nutrition transition in Brazil and the trajectory of Brazilian nutritional public policies, emphasizing the complexity of food problems and bringing the need for urgent reformulations to meet the new demand of obesity to the health system (Figure A1).

## 2. The Historical Context of the Nutrition Transition in Brazil

The work of Josué de Castro, entitled “Geography of Hunger”, generated available information to compose the first scenario of the food/nutritional problem in Brazil [8]. Escoda (2002) addressed the nutritional situation in Brazil in the last three decades, reporting that until the 1970s, the nutritional situation was strongly marked by epidemic outbreaks of hunger, geographically and socially located, with a high prevalence of severe forms of malnutrition [9]. Indeed, for many decades, the food consumption of Brazilians was inadequate, being characterized by a precarious diet, which is reflected in a significant number of people living in extreme poverty in the country [10]. Until the early 1970s, the profile of diseases in Brazil was associated with deficiency processes such as malnutrition (marasmus, kwashiorkor), anemia, and hypovitaminosis or infections resulting from inappropriate sanitary and hygiene conditions [11].

Only after 1975 were the inquiries effectively representative of the country’s nutritional situation, and differences between the macro-regions appeared in Brazil [8]. At that time, the nutritional situation was restricted to anthropometric data, excluding the state of calorie-protein malnutrition [8]. However, in the following decades, with the improvement of the inquiries, it was possible to analyze the evolutionary trends of the nutritional scenario in Brazil [8,10] (Table 1).

Interestingly, between 1975 and 1989, the epidemiological dynamics of malnutrition showed a unique evolutionary behavior, with a sharp decline in the problem when in all regions, except for the rural Northeast, the prevalence of cases of low body mass index (BMI < 18.5) became equivalent to the values found in developed countries, around 5% [8]. The change in this scenario was shaped by determining factors, such as declining fertility rates, a reduction in the pace of population growth, an increase in longevity, and increasing urbanization through the rural–urban migratory movement (populations that lived in the countryside and migrated to the large urban centers) [11]. These factors characterized the demographic transition and positively influenced the reduction in poverty and social exclusion in the country, consequently reducing hunger and universal access to food, especially industrialized foods [11].

In the 1990s, added to the problem of malnutrition, obesity rates increased [9]. Analyzing national studies from 1974/1975 and 1989, Monteiro (2000) observed a significant increase in overweight people in all age groups and social strata, overlapping malnutrition [12]. Corroborating with Monteiro (2000), the study of Batista Filho and Rissin (2003) cumulatively evaluated the prevalence of overweight and obesity among adult women and found an increase of 112% from 1974/1975 to 1995/1996 [8].

The increase in overweight and obesity rates in the Brazilian population was marked by the consumption of processed and ultra-processed foods [9,13]. As a result, there was an increase in saturated fatty acids, sugars, soft drinks, alcohol, industrialized products with excess “trans” fatty acids, meats, milk, fat-rich derivatives, and delicacies such as sweets, chocolates, and candies [9]. On the other hand, a considerable reduction in the consumption of complex carbohydrates, fruits, and vegetables was observed. This fact generated a picture of caloric excess due to the high intake of macronutrients (carbohydrates, proteins, and lipids) and micronutrient deficiencies (vitamins and minerals) [9]. Such changes in the dietary and nutritional patterns of the Brazilian population of all social strata and age groups culminated in nutrition transition, with a significant increase in overweight and obesity, increasing the development of non-communicable chronic diseases, and changing health/disease patterns, a process known as an epidemiological transition [11].

Recently, a national epidemiological survey showed a significant increase in the obesity rate from 20.8% in 2013 to 25.9% in 2019, with further rises between genders [14]. The more considerable increases were found in the 40–59 age group and the median income category for men. On the other hand, the highest increases were found among non-white women with low education [14]. In addition, recent data for 2020 demonstrated that the total costs of obesity in Brazil ranged from USD 133.8 million to USD 6.3 billion per year, varying from direct costs with healthcare and indirect costs in lower productivity and well-being [15].

The global dimensions of obesity demand efforts from international and national’s organizations to establish multisectoral strategies and recommendations from different governance levels [16] and the private sector [17]. The actual scenario indicates that the efforts to modify the unhealthy diet and physical inactivity environment might be challenging and set out the need for changes in the food system [18] and in economic and social policies [19,20].

## 3. The Trajectory of Food and Nutrition Public Policies in Brazil

Food and nutrition are determinant health conditions and are considered inherent rights for all people. The promotion and guarantee of adequate healthy food and nutrition have been mobilizing efforts from different sectors of the Brazilian government and civil society movements and entities [21,22]. Here we described the landmarks of the trajectory of the formulation and implementation of food and nutrition public policies in Brazil.

Despite the social inequities observed throughout the history of Brazil that culminated in food insecurity, only in the 1930s the problem of hunger entered the political agenda in Brazil, guided by the research of Josué de Castro [23]. De Castro observed that hunger and malnutrition were not natural facts but social phenomena [23].

Subsequently, studies of familiar budget and food consumption profiles ascended and were central to establishing the relationship between food and income. Such results contributed to the institution of the salary policy in 1936. It was believed that the minimum wage could guarantee a basic food basket with the necessary nutritional support for the laborers [24].

The landmark of food and nutrition policy was the creation of the Social Security Food Service (SSFS) in 1940. This policy optimized the access to food for the leading benefited population group: laborers. Its principal activities included providing meals, selling food at cost price, food and nutrition education actions, research support, and specialized technical training [25,26].

Still in the 1940s, other institutions, such as the National Food Technical Service (NFTS), 1942 to 1945; the Institute of Food Technology (IFT), 1944; and the National Food Commission (NFC), 1945 to 1972, were created to implement food and nutrition policies in Brazil, aiming the study of the country’s eating habits and nutritional situation. Right at the beginning, the NFC took “malnutrition” as the main topic of the agenda, assisting in elaborating the National Food Policy (NFP) with the Brazilian Federal Government. The NFC also created the National School Meal Program (1954) and improved the National School Feeding Program (NSFP) [21,25].

With the extinction of the NFC in 1972, the National Institute of Food and Nutrition (NIFN) was founded, operating until 1997. The NIFN directed the actions to population groups at risk or with nutritional deficiencies, such as pregnant women, nursing mothers, children, and workforces, through (1) food supplementation; (2) organization of the food production and marketing system; and (3) support social programs [25,27].

The NIFN was essential in promoting the National Food and Nutrition Program (NFNP), which unified the actions with others developed by institutions. The NFNP faced numerous difficulties and was extinct in 1974 [27]. After that, in 1975, the NIFN, supported by the Institute of Applied Economic Research (IAER), outlined the II NFNP, valid until 1979, that contributed to the creation of relevant programs for the food and nutrition area, including: (1) Worker’s Food Program; (2) Program for Rationalization of Basic Food Production; (3) Basic Food Supply Program in Low-Income Areas; (4) Food Supplementation Program; (5) Health Nutrition Program; (6) National Milk Program for Needy Children; (7) National Breastfeeding Incentive Program; (8) Food Supplementation Program; and (9) Programs for the Prevention and Combat of Specific Nutritional Deficiencies [27].

In the early 1990s, the National Food and Nutrition Surveillance System (NFNSS) was created. The purpose of NFNSS was to produce information that would allow the detection, description, and analysis of food and nutritional problems, identify the geographic and social factors involved, and support policies and procedures to prevent and correct these problems [25,26].

Another landmark was the implementation of the Unified Health System (Sistema Único de Saúde, SUS, in Portuguese) through Law number 8.080/1990, founded through the Federal Constitution of 1988 [28,29]. The central discourse of SUS was “Health is everyone’s right and a duty of the State”. Since then, the SUS has been contributing to the development of public policies in the field of food and nutrition, such as the foundation of the National Health Surveillance Agency (NHSA), which is responsible for creating norms and regulations and providing support for all sanitary control in the country, including the food sector [28].

In 1999, the National Policy on Food and Nutrition (NPFN) was approved, and the Ministry of Health declared the commitment to the eradication of consequences related to lack of food and poverty, especially children and maternal malnutrition, overweight, and obesity in the adult population [30]. Undeniably, until the end of the 1990s, the public policies in food and nutrition were more focused on hunger and malnutrition. However, the increase in the prevalence of obesity in Brazil evidenced the process of the nutritional and epidemiological transition experienced by Brazilians [31]. Thus, modestly, the Ministry of Health started the implementation of actions for the health promotion of individuals with obesity [32].

As of 2004, the priority on the political agenda was to strengthen the discussion on food and nutrition security (FNS), first with the reopening of the National Food and Nutrition Security Council (NFNS) and with the approval of the Organic Law on Food and Nutrition Security (OLFNS) in 2006, which instituted the creation of the National Food and Nutrition Security System (NFNSS) [33,34]. Then, the insertion of the Human Right to Adequate Food in article 6 of the Federal Constitution in 2010 was consolidated as a significant milestone, guaranteeing physical and economic access to adequate food or obtaining it [35]. Among the various intersectoral actions, Law 11.947 stands out, which regulates food from the NSFP, associated with the purchase of food from family farming [36,37].

The program “Intersectoral Strategies for the Prevention and Control of Obesity” of the Ministry of Health, created between 2010 and 2014 [32], disclosed obesity as a social problem and implemented six axes of action for the promotion of health care for individuals with obesity, being them: (1) availability and access to adequate and healthy food; (2) education, communication, and information actions; (3) promotion of healthy lifestyles in specific environments; (4) food and nutrition surveillance; (5) comprehensive health care for individuals with obesity in the health network; and (6) regulation and control of food quality and safety [32]. The promotion of adequate and healthy food is one of the components of health promotion and encompasses actions of encouragement, support, and protection. These actions aimed to disseminate information, facilitate, and protect adherence to healthy eating practices, such as mandatory nutrition labeling, food guides, and the regulation of food advertising [38]. In addition to healthy eating, some documents adopt the concept of adequate and healthy food, dialoguing with the food and nutrition security policy, which refers to the cultural, social, and economic adequacy of food, not just nutritional [38]. The document entitled “Primary Care Book-Strategies for the care of people with chronic disease: obesity” is an example of the government action to stimulate the healthy eating, based on the creation of strategies to approach the disease and the allegation of various behaviors and consequences arising from obesity [32]. The “Food Guide for the Brazilian Population” is another government action part of the NPFN. This food guide serves as an instrument to support and encourage healthy eating practices at the individual and collective level, as well as to subsidize policies, programs, and actions that aim to encourage, support, protect and promote the health and FNS of the population [39].

## 4. Current Status of Knowledge of Obesity’s Management and Public Nutrition Policies in Brazil

Several initiatives have been developed in Brazil to deal with the challenge of reversing the rising overweight and obesity rates and reducing their impact on the population’s disease burden and quality of life [40]. However, the analysis of health survey data indicated temporal trends of accentuated growth in the prevalence of overweight and obesity in Brazil, indicating fallacies in the implementation of food and nutrition public policies. Dias et al., 2017 pointed out that the public policies focused on biomedical interventions traditionally limited to the biological dimension and treatment of obesity once it already exists proved not to be effective in reducing its prevalence. Individual or group consultations, bariatric surgery, and pharmacological interventions, even if they could be mass-produced, would not be sufficient to attack the problem’s main conditioning factors. In addition, adherence to individualized treatment is low because individuals are still exposed to the same environmental pressures that compete unequally with personal motivation to modify eating habits and behaviors. The author proposed measures based on the social/environmental health approach, aimed at guaranteeing healthy environments and life settings, which may potentially favor proposals for inter-sector linkage through such measures as guaranteed access to adequate and healthy eating [41].

A recent study by Lopes et al., 2021, investigated the adequacy of basic health units in Brazil regarding structure and work process for obesity management in primary health care (PHC), using data from the National Program for Improving Primary Care Access and Quality. They evaluated three indicators: (i) adequate structure-equipment, human resources, and facilities; (ii) adequate access-available collective and individual healthcare interventions; and (iii) adequate service organization and management-systematization of procedures, adequate use of medical records, matrix support, and permanent education. They found that 7.6% of PCH units presented an adequate structure, only 26.6% of the units presented adequate access to obesity care, and 27.8% were considered to have an adequate service organization. According to the authors, services structure and available materials tend to affect the resolution of PHC actions. In addition, considering that more than 3/4 of the population exclusively uses the public health system and that more than 1 million Brazilians have severe obesity, the lack of adequate structure for diagnosing, evaluating, and monitoring obesity may lead to poorer quality care, increased risk of obesity-related complications and, consequently, increased healthcare spending [42].

Brazil has made commitments with the United Nations to halt the growth of obesity among adults, with a reduction in the consumption of sugary drinks, an increase in the consumption of fruits and vegetables, and a reduction in the consumption of ultra-processed foods. In this sense, Brazil’s National Health Surveillance Agency (NHSA) published a proposal in 2006 to regulate the advertising of foods high in sugar, sodium, saturated fat, and trans-fat in the form of public consultation. The public hearing to present these regulations was held in August 2009 (with strong participation of civil society groups, members of the academic community, and representatives of the food retailers, manufacturers, and media marketing groups involved), and the final text was published on 15 June 2012. The resolution aimed to ensure access to health information for all to curb excessive practices that lead the public (especially children) to adopt eating habits that are incompatible with health and infringe upon their right to an appropriate diet. The resolution was challenged in court by several lawsuits opened by different sectors and associations (most of which related to the food industry) and was suspended by the federal attorney general despite the commitment of the Health Ministry and the NHSA (supported by social control representatives, such as consumer associations and universities) [40]. The Brazilian experience regarding the regulation of food advertising points to the need to build a broad political consensus on this subject among the government’s executive, legislative and judicial branches, along with social support and acceptance [40].

Interestingly, Passos et al., 2020 investigated the relationship between the price of ultra-processed foods and the prevalence of obesity in Brazil, based on data from the National Household Budget Survey from 2008/09 (*n* = 55,570 households, divided into 550 strata). They concluded that the price of ultra-processed foods was inversely associated with the prevalence of overweight and obesity in Brazil: for each 1.00% increase in the price of ultra-processed foods, there was a mean 0.33% decrease in the prevalence of overweight and of 0.59% in that of obesity. The author highlighted that the taxation of ultra-processed foods emerges as a prominent tool in the control of obesity [43].

Regardless of the limitations of public policies implementation, Fernandes et al., 2019, highlighted the innovations presented in the new food- and meal-based Dietary Guidelines for the Brazilian Population, especially the mention of the preference for natural or minimally processed foods and freshly made dishes and meals to ultra-processed products, and the encouragement to culinary and cooking skills. All these recommendations are in accordance with the World Health Organization. In addition, the authors emphasized that public health nutrition policies should be considered as a part of an individual’s right to information about food. Consumers can choose to make healthy choices if they have access to information, comprehend nutritional information, and healthy food preparation is available [44].

## 5. Conclusions

The historical progress of the nutrition transition in Brazil interweaves with the trajectory of Brazilian nutritional public policies, emphasizing the complexity of food problems and bringing the need for urgent reformulations to meet this new demand. Our review suggested that the country may still be at the initial stage of care for obesity, and more efforts are needed to contain the advance of the disease in Brazil. In this sense, we highlighted the importance of the government’s commitment to health in various spheres, such as access to adequate and healthy food, social assurance, and sustainability. We also underscored the importance of the nutritional public policies for nutritional surveillance, promotion of health, healthy habits, and primary and comprehensive care for all individuals: from malnutrition to obesity.

## Figures and Tables

**Table 1:** Evolutionary trends of the nutritional situation of men and women in Brazil, according to data obtained in national surveys.

	Nutritional Situation		
Inquiry (Year)	Underweight	Overweight	Obesity
**Men**			
NSHE (1974–1975)	8.0%	18.5%	2.8%
NSHN (1989)	4.4%	29.9%	5.4%
HBS (2002–2003)	3.1%	41.4%	9.0%
HBS (2008–2009)	1.8%	50.1%	12.4%
NHS (2013)	2.1%	55.5%	16.8%
NHS (2019)	1.7%	57.5%	21.8%
**Women**			
NSHE (1974–1975)	11.8%	28.7%	8.0%
NSHN (1989)	6.4%	41.4%	13.2%
HBS (2002–2003)	5.6%	40.9%	13.5%
HBS (2008–2009)	3.6%	48.0%	16.9%
NHS (2013)	2.8%	58.2%	24.4%
NHS (2019)	1.5%	62.6%	29.5%

Note: NSHE: National Survey on Household Expenses; NSHN: National Survey on Health and Nutrition; HBS: Household Budget Survey; NHS: National Health Survey.
